# Mental health and psychosocial problems among conflict-affected children in Kachin State, Myanmar: a qualitative study

**DOI:** 10.1186/s13031-018-0175-8

**Published:** 2018-09-19

**Authors:** Catherine Lee, Amanda J. Nguyen, Tara Russell, Yasmina Aules, Paul Bolton

**Affiliations:** 1 Department of International Health, Johns Hopkins School of Public Health, 614 N. Wolfe Street, Baltimore, MD 21205 USA; 2 Department of Human Services, Curry School of Education, University of Virginia, 405 Emmet St S., Charlottesville, VA USA; 3 Independent Contractor with the Applied Mental Health Research group, Johns Hopkins School of Public Health, Baltimore, MD USA; 4 Center for Refugee and Disaster Response, Department of International Health, Johns Hopkins School of Public Health, Baltimore, MD USA; 5 Department of Mental Health, Johns Hopkins School of Public Health, Baltimore, MD USA

**Keywords:** Myanmar, Mental health, Qualitative, Conflict, Internally displaced persons, Child, Adolescent, Youth

## Abstract

**Background:**

In Kachin State, Myanmar, collapse of a ceasefire in 2011 has resulted in widespread exposure to conflict and ongoing internal displacement. Such exposures are known risk factors for mental health and psychosocial (MHPS) problems, yet mental health services for children and youth are typically scarce in such circumstances. Following evaluation of a mental health treatment for adult trauma survivors on the Thailand-Myanmar border, our study team received requests to support the development of a similar intervention for displaced children in Kachin State. To inform this work, we conducted a brief qualitative needs assessment to explore priority MHPS problems among this population.

**Methods:**

Data were collected in internally displaced persons camps in Kachin State during July and August, 2016. Free list interviews with a convenience sample of 28 adolescents and 12 adults produced a list of problems affecting children and adolescents in this area. Four problems were further explored in key informant interviews with a convenience sample of 26 adolescents and 4 adults. Data analysis was conducted by the local interview team.

**Results:**

Priority problems included: behavior problems, substance use, effects of war, and feeling sad/depressed/hopeless. Descriptions emphasized the interconnectedness between the problems. Overall, most problems were related to specific events that suggest that the symptoms themselves are responses to unusual situations; however, the problems were also linked to current psychosocial stressors such as poverty, poor nutrition, and discrimination. Effects of war were described primarily as a constellation of social and economic problems rather than a list of mental health symptoms, although descriptions of these problems did include post-traumatic stress symptoms.

**Conclusions:**

Findings fit well within explanatory models of distress that include both direct trauma exposure and exacerbation of daily stressors. Results of this study have been used to inform intervention adaptation and evaluation, but also contribute to the literature on the needs of young people in situations of protracted conflict.

## Background

Children in armed conflict experience both direct consequences of violence such as of unlawful recruitment into armed forces, killings, gender-based violence, trafficking, and illegal detentions, separation from families, forced displacement, etc., as well as indirect impacts such as lack of access to basic services and increased poverty, malnutrition, and disease [1]. The mental health consequences of conflict on children are clear, with elevated rates of post-traumatic stress, depression, and anxiety in conflict affected children [2]. These impacts are recognized as being due to both direct exposure to traumatic events as well as exposure to increased levels of daily stressors [3]. However, availability of mental health interventions for conflict-affected children is lacking, with treatment gaps for children even higher than those for adults in low resource settings [4], and even those services available have typically not been rigorously evaluated [5, 6]. Barriers to improving access to child mental health services in low resource settings include not only the lack of evidence for treatments and shortage of skilled professionals, but also low recognition/detection of mental disorders among children [7], highlighting the need to improve our understanding of how mental health problems present among children - particularly in socioculturally diverse areas where child mental health has rarely been studied.

In Myanmar, internal civil and ethnic conflicts have resulted in long-term displacement of children with documented impacts on their mental health and wellbeing [8, 9]. One of the main conflicts in Myanmar is in Kachin State where fighting between the Kachin Independence Army (KIA) and government soldiers has been ongoing since 1961. In 2011, a 17-year ceasefire agreement between these two groups collapsed when government forces attacked KIA positions to seize KIA-controlled areas around the sites of key energy development projects. In 2012, fighting escalated with ground conflict along the main transportation route, the Myitkyina-Bhamo road, and airstrikes launched by government forces continuing in to 2014. The conflict between the KIA and government forces has resulted in civilians experiencing widespread exposure to conflict and ongoing internal displacement. Over 119,000 people in Kachin state and neighboring Shan state continue to need humanitarian assistance, including over 98,000 displaced as a direct result of the conflict since the end of the ceasefire [10]. Rural families have been disproportionately affected and relocated to internally displaced persons (IDP) camps situated in or near urban areas. Camp residents rely primarily on support from church group donations and live in basic housing that is often made of bamboo and simple wood structures, sometimes housing eight to ten extended family members together. Camps tend to be close to or within the compound of a host church and although there is a curfew, residents can come and go freely from the camp. Adults and some adolescents work as day laborers to earn supplemental income. and children typically attend school either within the camp or at nearby government schools when a school is not available within the camp.

The on-going conflict and protracted displacement have resulted in significant impacts on livelihoods at the household level, which in turn have resulted in concerns regarding access to food, medical and basic services, and being generally protected from the effects of the fighting. In addition, there have been documented increases in social problems including, but not limited to, sexual and gender-based violence perpetrated by government military, drug use and abuse, forced recruitment into armed service conducted by both sides of the conflict, forced labor for construction, maintenance and servicing of military camps or other work to support the government military, lack of access to health services, exploitation and human trafficking [11]. These protection concerns can affect the populations in Kachin state both during displacement and once settled in camps for internally displaced persons. Mental health and psychosocial programs (MHPSS) are limited in Myanmar, particularly in ethnic states such as Kachin state that continue to experience conflict between armed groups and the government military. Key limitations to the MHPSS services in Kachin state are limited technical assistance, lack of referral for higher level care, and, ultimately, a lack of a community-based interventions with documented effectiveness [12], particularly for children and adolescents.

### Current study

We previously developed and evaluated a mental health intervention – the Common Elements Treatment Approach (CETA) [13] – along the Thailand-Myanmar border that was found to be acceptable, accessible, and effective in improving mental health and functioning of adult trauma survivors from Myanmar [14]. During scale-up efforts following the initial trial we received feedback on the need for similar services for children in Myanmar, and requests from community-based organizations to implement CETA for children in Kachin State.

Because our previous work in Myanmar focused on adults from a different ethnic group in a different area of the country, the purpose of the current study was to qualitatively assess the priority psychosocial problems affecting displaced children and adolescents in Kachin State. This research served as a necessary first step to determine whether CETA would be an appropriate intervention to meet the needs of this population, and to inform the adaptation and evaluation of the intervention. These findings also serve a broader aim to improve our understanding of the mental health problems and needs of young people in the context of prolonged and on-going displacement and conflict.

## Methods

### Study design

This study was conducted in partnership with two local organizations currently providing basic psychosocial support services to IDP in Kachin State: Kachin Baptist Convention (KBC) and Kachin Development Group (KDG). The study design was based on the JHU Applied Mental Health Research Group’s Design, Implementation, Monitoring, and Evaluation (DIME) research model [15], Module 1, which has been used in many settings with both children and adults to elicit locally defined mental health and psychosocial problems [16–21]. This approach draws on two primary methods of data collection: first, brief free list (FL) interviews are conducted to develop a list of problems affecting the target population. From this list, priority problems are selected for further exploration in in-depth key informant (KI) interviews.

### Setting

Data were collected in Myitkyina and Laiza, two cities located in Kachin State in northeastern Myanmar, in July and August, 2016. These sites were chosen because they are the areas where the two local partner organizations, KBC and KDG, provide services and where they planned to implement mental health counseling services. Myitkyina is the capital city of Kachin state and is situated just over 100 km northwest of Laiza, a town on the border with China that is also a central base for the KIA. Myitkyina is a larger, urban city compared to Laiza, which is comparatively smaller town in a more remote location. Specific townships within Myitkyina city limits (noted below) were selected due to the presence of IDP camps in these areas. FL interviews were conducted in both locations. Both partner organizations then limited KI interviews to Myitkyina due to communication and security problems in the Laiza area. The sites for data collection were similar in that populations in the sites were all directly impacted by the conflict and of the same ethnicity, Kachin.

### Participants

Study participants were adolescents and adults who were recruited using existing contacts of the two local partner organizations based on exposure to conflict, violence, and other types of trauma.

FL respondents were a convenience sample from six IDP camps in or near Myitkyina and Laiza. For all participants, inclusion criteria for participation was that individuals must have, themselves, experienced conflict, violence, and/or torture or be deemed knowledgeable enough about the impacts of these experiences on individuals, specifically children and adolescents. Populations not living in Kachin state, Myanmar were excluded. In addition, adults and adolescents not able to provide informed consent/assent due to mental disability were excluded, such as those with active psychosis or serious developmental disorders that would preclude participation in an interview. The inclusion criteria allowed for youth aged 10 years and above to be included, however, the actual sample included adolescents 12–17 years of age (*n* = 28). Adults considered to be knowledgeable about the problems of children and adolescents (*n* = 12) were also interviewed for the FL activity. These adults were identified by the local partner organizations and were parents, teachers, or other adults who were involved in providing services to children and adolescents. We purposively sampled to have a balance in gender for respondents and to capture a range of ages (12–17 years for adolescents, mean = 14.7 years; 18–60 years for adults, mean = 34.6 years). Of the 40 free-list interviewees, 23 were female and 17 were male.

In-depth KI interviews were also conducted with a convenience sample of respondents in three IDP camps in or near Myitkyina. and included adolescents 10–17 years of age (*n* = 26) and adults knowledgeable about the problems of children and adolescents (*n* = 4). Key informants were selected by staff from the local community based organizations operating in the IDP camps with assistance from camp authorities. These staff and camp authorities were asked to identify potential participants who were knowledgeable about the problems of children and adolescents affected by conflict, trauma, and violence, including youth themselves as well as teachers, parents and church members who organize activities for children. In all cases, the key informants were part of the local community and were knowledgeable about the problems of conflict, violence and other trauma but did not deal with these problems professionally (i.e. were not professional health care workers, social workers or counselors). Professionals were excluded because they will tend to answer based on their training rather than reflect on local community and cultural perspectives.

Of the 30 key informants interviewed, 14 were female and 16 were male.

### Data collection

For FL interviews, a team of eight local data collectors were trained in research ethics, qualitative methods, and data analysis. For KI interviews, four of the original data collectors received refresher training on research ethics, qualitative methods, and data analysis, with additional information about conducting in-depth interviews. The study lead (CL) prepared the training materials and oversaw the work of the Research Director (YA) who carried out on-site activities. The Research Director had experience leading training, data collection supervision, and analysis processes for three previous qualitative studies using similar methods as this project with populations in Myanmar. Interviewers were staff from the Kachin Development Group (*n* = 4 for FL interviews) or Kachin Baptist Convention (*n* = 4 for FL and KI interviews). The FL interviewers included one male and seven female interviewers who ranged in age from 18 to 55 years (mean = 25.5 years). Their existing professions included being a volunteer data entry staff or data collector for the organization, a teacher, a malaria control program staff person, or youth leader. The interviewers who conducted KI interviews included one male and three female interviewers ranging in age from 18 to 55 years (mean = 29.8 years).

Interviewers worked in teams of two to carry out the data collection and record interviews in the local Kachin language (Jingpaw). Within each pair, one person acted mainly as the interviewer and the other mainly as the note taker. Interviews normally lasted 1 hour or less. Following each interview, the interviewer and note taker would compare notes to ensure that the most complete transcription of the respondent comments was recorded.

Free list interviews involved asking respondents to provide lists of items (and brief descriptions of each item) in response to two questions regarding: 1) all the problems facing children and adolescents ages 6–17 years in the community; 2) how these problems affect families. For each question, interviewers probed respondents until no more problems or activities were listed.

Following the analysis of FL data (described below), KI interviews were conducted to gather more in-depth information on the priority psychosocial problems selected from the FL data. For the KI interviews, interviewers asked respondents to describe five domain for each of the problems: a) the nature of the problem, including a description of symptoms and effects; b) the cause of the problem; c) effects of the problem for the individual, family, and community; d) what people currently do about the problem, and; e) what they think could or should be done about the problem. Interviewers probed at the end of each question on whether there were important differences between younger children (6–11 years old) and adolescents (12–17 year old).

### Ethics

Respondents were read a standard information sheet about the study. Adult respondents and adult caregivers gave verbal informed consent and adolescents under age 18 gave verbal assent to be interviewed for the study. All individuals approached agreed to participate in the study.

Names and contact information for potential respondents were recorded in a notebook separate from data. After agreeing to participate in the study an identification number was given to each respondent. This identification number was linked only with age, sex, and marital status of each respondent. Names and contact information kept in the separate notebook were destroyed after the interview was completed.

For both types of interviews, the questions asked about problems affecting children and adolescents in general and we did not seek information about the specific problems of individual respondents. Interviewers were trained to redirect any discussion about personal experience or problems to focus back on the problems of children and adolescents in the community in general. Interviewers were also trained to stop an interview and contact his or her supervisor if a respondent became upset; however, during data collection there were not instances of a respondent becoming upset and a supervisor needing to be contacted. The supervisors were program coordinators from each of the organizations and were present and available on site during both data collection activities. Both the JHU Institutional Review Board (IRB: 6933) and the Department of Medical Research Ethical Review Board in Yangon, Myanmar (IRB: 001116) reviewed and approved this study protocol.

### Analysis

The interviewers and the JHU Research Director conducted analysis of the free list interviews in Kachin language with oral translation to Myanmar language for the Research Director provided in real time by a certified translator working as the JHU Assistant Research Coordinator. The first step of the analysis consisted of reviewing all responses to the two free lists of problem descriptions. Interviewers listed all response items and problems along with the brief descriptions for each. When items were worded differently, the group made decisions by consensus as to whether or not the items were the same or distinct from each other. If the group agreed that two items were the same, they selected the more appropriate wording to include on the list; if the group could not agree then both items were listed as separate problems. The resulting lists of problems were then ordered by frequency of reporting. The group then discussed the various brief descriptions to come up with a single brief description for each item using the language spoken by respondents that best described the problem. The final lists were typed in Kachin and then translated into English.

The study team then reviewed the final lists and identified problems about which more detailed information would be sought using KI interviews. Selected problems were those which: a) were not yet well understood; b) could feasibly form a focus for an evidence-based intervention provided by local workers without prior mental health experience using available program resources; c) were mentioned by many respondents, and; d) appeared to be severe, based on the description of the problem and what was currently known about it.

As in the free list analysis, the interviewers and JHU Research Director worked in Kachin and Myanmar languages to review all KI interview transcripts and recorded responses relevant to the individual problems. The group determined categories of responses under individual questions through group discussion, with full quotes pulled from the transcripts to retain specific wording and information given by respondents. When a category was determined, the group decided by consensus which phrase was described the overall category, however, in some cases several phrases were retained to capture the breadth of the information. A final table was produced including all responses and the number of the KIs who gave each response. Analysis tables were generated in electronic format in Kachin and then translated to English in typed format by the same translator who conducted oral translation for the Research Director during analysis.

## Results

### Free lists

Table 1 shows the full list of problems affecting individuals mentioned by three or more respondents, as well the number of adult respondents for each problem. Comparison of the adolescent and adult data from the free list interviews shows similarities in the frequency of problems. In addition, comparison of the responses by site found that frequency of reported problems varied little and all problems were mentioned by at three or more respondents were mentioned by at least one person from each site. From the free list interviews, numerous behavior issues were mentioned by the respondents such as gambling, stealing, bullying, fighting, and other kinds of misbehaving by children and adolescents. The research team decided to describe these issues as “Behavior Problems” and cover all of them in one question in the key informant interviews. Several “substance abuse” and “effects of conflict” references were also reported in the free lists and deemed relevant to be explored as two additional problems in the key informant interviews as it was thought that “substance abuse” and “effects of conflict” problems would raise other mental health issues. “Sad/depression/hopeless” were feelings frequently expressed by the free list respondents and included as the fourth question for key informant interviews.

**Table 1:** Problems affecting children and adolescents (40 Free List Respondents)

Problems Affecting Individuals	Number^a^	Among Adults^b^
Dropping out of school	30	8
Drug abuse	27	8
Alcoholism	20	6
Lack of money	19	5
Unwanted pregnancy	17	5
Unhealthy	16	4
Stealing	14	3
Early marriage	14	4
Quarreling	13	2
Not enough space (for living)	12	5
Fled from war	11	3
Human trafficking	11	4
Having love affair	10	3
Child labor	10	5
Disagreement in the family	8	4
Misbehaving of children	8	4
Travel difficulty	7	3
Lack of child rights	6	3
Rape	6	4
Malnutrition	4	3
Lack of basic things	4	0
Abortion	4	1
Children unattended	3	1
Lack of faith	3	2
Staying at boarding houses	3	1
Gambling	3	0
Suicide	3	2

^a^Responses given by two or fewer respondents are excluded

^b^Out of a total of 12 adult respondents

### Key informant interviews

Below we summarize the key themes frequently reported for each of the selected problems. As probing about important differences in the experience of these problems between younger children and adolescents identified no reported distinctions, the provided descriptions are applicable to both age groups. In addition, comparison of responses between adolescents and adults found no significant variation.

The most frequently listed **behavior problems** (Table 2) were arguing, stealing, bullying, quarrelling (physical fights), skipping school, and lying or swearing. The description of the causes of arguments, stealing, and skipping school seem related to low financial resources of parents in that arguments and stealing were a result of not getting things children want because their parents cannot afford it or parents being unable to afford school fees and skipping school was sometimes a result of parents not being able to afford school fees so children would skip school to earn or steal their own income. Environmental influences such as movies, peers, and family were mentioned in relation to many of the behavior problems and explained as being caused by “watching movies, seeing parents do this, and older siblings bullying them at home… so they bully other people and feel happy bullying others”. Although skipping school was related to parents’ inability to pay school fees, it was also explained as being caused by a “desire for truancy and wanting to go to the gaming station (gambling) or swim at the stream and avoid being beaten by teachers when they are already behind in school lessons.”

**Table 2:** Problems related to “Behavior problems” (30 Key Informants)

Topic-Behavior problems	Number^a^
Arguing	25
Stealing	20
Bullying	20
Quarrelling	20
Skipping school	18
Lying and swearing	16
Going to game station	9
Wandering around	8
Courtship	6

^a^Responses given by two or fewer respondents are excluded

The negative impacts of these behavior problems on individuals centered mostly around physical punishment from both parents and teachers, specifically described as results of arguing, quarrelling, bullying, skipping school, and lying or swearing. In response to quarrelling children were “being beaten by parents and face being sent away from the house”. “Being beaten by teachers for not understanding the lessons” was also listed as a negative impact and a cause of skipping school. It is unclear if physical punishment from parents and teachers has increased due to the political violence in Kachin state, however, in Kachin state and across Myanmar, corporeal punishment is legal and common in homes and schools. Regarding the negative impacts of bullying, a child might start to bully others because they are bullied and this could lead to “suffering dislike from others, gradually losing friends” and experiencing not only physical pain but “psychological pain” as well.

The impacts of behavior problems on families included fighting between parents or parents feeling ashamed that their child is exhibiting these behaviors. For lying and swearing, quarrelling, stealing, and bullying: each of these would result in parents being criticized and judged by others or socially isolated from others in the community because they are blamed for their child’s behavior. Stealing and going to the gaming station or gambling had negative financial impacts “causing the family to lose money” and creating financial hardship because “parents have to compensate and make up for what is stolen”. Impacts on the community because of behavior problems were described similarly to impacts on the family, with a central theme being that communities shun children with behavior problems and their families. “Parents worry that their child will behave like this…so they do not allow visits to the house where children are bullied” and “they are afraid their children will follow neighbor child’s bad behavior”. These bad behaviors were said to cause unhappiness in communities, increase worry about security in the camps, and may “ruin the reputation of the people in the camp”, particularly with regard to stealing, quarrelling, and gambling.

**Substance abuse** (Table 3) problems were described as abuse of a variety of substances including: methamphetamine, tobacco, heroin, glue-sniffing, and alcohol. The signs of substance abuse included “seeing that they sleep the whole day and are unconscious all of the time without being aware of it being day or night” as well as seeing children physically use these substances or have traces (smell, track marks) of use. Influences from friends, peers, and parents, separation from parents and lack of supervision from parents, as well as easy access to and low cost of the substances were explained as a cause of all types of substance abuse listed. In addition, it was explained that children and adolescents try or abuse these substances out of curiosity. Specifically related to mental health, respondents explained that methamphetamine is abused because a child “lacks dreams” and that children sniff glue or drink alcohol because they “feel depressed”. Other signs of substance abuse included feeling sad, anxious or having difficulty maintaining friendships or control over one’s emotions.

**Table 3:** Problems related to “Substance abuse” (30 Key Informants)

Topic-Substance abuse	Number
Methamphetamine (“Yaba” / Dextro)	29
Tobacco	24
Black Heroin (opium), White Heroin (heroin)	24
Glue-sniffing	23
Alcohol	22
Betel	20

Negative impacts of substance abuse were frequently described as failing in school or dropping out of school, as well as becoming physically sick, and getting “psychological diseases”. It was also explained that children who use substances may be physically beaten by parents. Relationships with community and family were also negatively impacted because children who abuse drugs are “dissociated from the community” and “alienated by friends”.

The family impact of substance abuse among children and adolescents was commonly described as causing family problems that include arguments between parents, distancing from relatives, lose of money, and stress within a family. As a result, substance abuse then created further problems for communities by increasing gossiping about or criticism of these parents and families and generating worry for the community because “no educated people are generated if they drop out of school” and general worry about the future of the community. Respondents further explained that “we cannot sleep well at night because we worry they will steal our property and we have to be alert… it spoils the society’s reputation” and they commented on disintegration of society and general community insecurity and anxiety because of substance abuse by children and adolescents.

**Effects of conflict** (Table 4) and resulting problems include school drop out, food insecurity, economic problems, shelter insecurity, separation from family, as well as disability and torture. Separation from family was described as resulting from both economic hardships where a parent or parents would need to live and work in a place different from the child and because of death of one or both family members and a child is sent to live with others. Substance abuse was listed as a result of experiencing the effects of conflict and was described in relation to school drop out and economic problems within the family. Further, within the descriptions of the overarching problems, respondents described mental health and psychosocial impacts on children and adolescents that were seen as a direct result of having experienced conflict, such as: behavior problems (stealing, lying, fighting with others, bullying), depression and sadness, worry, distress, blame others, feeling insecure and unsafe, easy to anger, afraid, shock, and crying all the time. Respondents from Myitkyina explained that children and adolescents affected by conflict often were looked down upon by others (peers, community) and, as a result, felt embarrassment and shame. This was often explained as being a result of resulting poverty from the conflict, but was also related to having dropped out of school and to being orphaned from the conflict. For IDP living in Myiktyina, interaction with non-IDP populations is common because the camps in which they live are located at churches in Myitkyina and youth attend school with non-IDP peers, which means they are more frequently in contact with non-IDP populations. In contrast, the entire population in Laiza can be considered as conflict-affected because this is not a government controlled area. Attempted suicide was also discussed in relation to having seen or experienced torture during the conflict.

**Table 4:** Problems related to “Sad/Depression/Hopeless” (30 Key Informants)

Topic- Sad/Depression/Hopeless	Number^a^
Being sad	16
Self-centered	10
Gazing	9
Crying	7
Feeling depression	7
Being frightened	3

^a^Responses given by two or fewer respondents are excluded

Negative impacts on families of children experiencing these problems as a result of the conflict included increased fighting among family members, parents worrying about their children, physical illness due to poor nutrition, depression and sadness among all family members, and social isolation from others in the community. For all of the problems, respondents reported a general widespread impact on the feelings of family members and disruption of family relationships as explained by one respondent with regard to shelter insecurity: “families cannot find happiness or think logically…they have poor sleep and unity of the family collapses because they get angry with each other easily and fight amongst themselves”.

Community impacts were similar with descriptions of responses to school drop out being criticism and alienation of the child who dropped out of school or his or her family. Food insecurity also resulted in increases of theft in a community, which in turn was described to result in causing unhappiness and general trouble in the community – despite some respondents expressing sympathy for those who steal because of food insecurity and a desire to be charitable towards them. This type of response was also given in relation to shelter insecurity, disability, death of a parent or family member, and separation from family. However, community responses to those experiencing economic hardships were described as being more similarly aligned with response to those who drop out of school and included community members looking down upon or exploiting the children or families with children who experience poverty as a result of the conflict.

**Sad, depression, hopeless** (Table 5) was described as typical representations of a combination of trauma symptoms and symptoms of depression. Common signs of a young person feeling sad, depressed, or hopeless included: crying, eating meals irregularly, not playing with friends, staring or gazing, being unwilling to talk with people, staying alone, sleeping all of the time, not visiting neighbors, and being frightened when hearing sounds. Low school attendance or no attendance at school was also a result of sadness as explained in this domain. The concept of being “self-centered” was explained as not talking with others and this was further elaborated and explained as being withdrawn from others “because of leaving their own house…the booming sounds and military aircraft, seeing people shot, seeing rape, and feeling day and night”. In general, the causes explained under “sad, depression, hopeless” were physical displacement from their homes, witnessing shootings, combat and rape, and witnessing torture. In addition, all of the effects of sadness and hopelessness listed above were explained as being caused by separation from family or becoming an orphan, in some cases even witnessing the death of their parents.

**Table 5:** Problems related to “Effects of conflict” (30 Key Informants)

Topic- Effects of conflict	Number^a^
Dropping out of school	23
Not enough food and money	18
Economic recession	16
Lack of shelters and not enough space to live	15
Disabilities	11
Seeing death	10
Separation from family	10
Torture	7

^a^Responses given by two or fewer respondents are excluded

Sadness and hopelessness were described as resulting in both physical and mental health problems. These included loss of appetite, stomachaches, sleeping poorly, being angry, lacking concentration, feeling hopeless, unable to connect with or communicate with others, feeling heart pain, and “worrying so much that it results in disease of the heart”. Attempted suicide was also discussed as an impact of feeling sad and hopeless and was linked by respondents to a loss of hope for their future and lack of a future because of missing out on educational opportunities. This cycle of the connection between mental and physical problems and the resulting impacts on a young person’s life was explained as: “they suffer stomachaches, psychological problems, and other diseases because of loss of appetite and not being able to concentrate…when they cannot concentrate they do poorly in school and this can lead to them becoming pregnant at a young age.”

The impact on families when children and adolescents have feelings of sadness, depression, and hopelessness were described as creating worry among parents, fighting between parents, conflict between family members, being “looked down upon by others and called a ‘crazy family’ by others in the community”. It was also explained that parents would also feel depression when they saw their children feeling depressed, which in turn reduced the ability of the parents to work for income and, at times, resulted in attempted suicide by the parents.

Negative impacts on the community as a result of this problem were frequently explained to include: feeling sorry for the children, losing sleep because of worry for the children, and in some cases causing trouble between neighbors because a child in the community is experiencing depression.

#### Support and coping

Respondents commented on a number of existing support systems and coping mechanisms for the above-listed problems. Across the four main problems, the current role of adults in the community was mentioned. Specifically, respondents commented on community and community-based organization support for children and families experiencing food security. In addition, camp leaders, parents, church community and religious leaders were mentioned as current support for children experiencing the four problems. It was explained that these adults attempt to provide intervention, encouragement, support, and prayers. Respondents specifically commented that children experiencing these problems are, for the most part, receptive of these interventions, particularly when the information comes from religious leaders or church groups. Teachers, however, were only mentioned regarding providing advice and guidance on behavior problems.

Self coping mechanisms for children experiencing these problems included “controlling one’s own mind”, problem solving by one’s self, making new friendships with “good friends”, playing and spending time with friends to forget about the problems (specifically for sadness and depression), singing songs, listening to music, and sharing feelings with people close to them. For the problem of economic hardship as a result of experiencing conflict, coping mechanisms included learning a specific vocation through training, finding a job as a day laborer, in gold mining, cleaning, cooking, washing dishes at a shop, or farming rice. When asked what should or could be done to support children with these problems or ways that children with these problems should cope, the responses were very similar to current support and coping mechanisms, thus there were no suggestions for new approaches.

For all problems, parents, religious organizations and leaders, and camp committee leaders were all mentioned as people that more support could be provided. This support, adolescent and adult respondents suggested, could be given through encouragement, prayers, youth-specific programs (specifically bible and religious studies), and taking specific action on the problems. These specific actions were explained as possibly including limiting sale of alcohol in the camps, intervening to deal with behavior problems, providing support in the form of food or cash assistance for those experiencing poverty as a result of displacement from the conflict, and giving advice about positive coping strategies for sadness. However, details on new strategies were not reported by respondents. Respondents also acknowledged that additional support should be given to parents of children experiencing these problems. Specifically, they responded that the community or organizations should help parents pay school fees so children can stay in school, provide food support, help with home repairs for those with shelter insecurity, give medicine and other necessary household supplies, and, finally, to give them workshops on how to effectively deal with behavior problems their children might be exhibiting.

In addition to continuing the current positive coping mechanisms children do already in response to these problems, respondents added that there could be more organized events for children such as games, storytelling, creating and letting them spend time on playgrounds with friends, and having retreats for children to get time away from the camp.

## Discussion

Using brief, qualitative methods, the current study identified and explored four priority MHPSS problems affecting children and young people in Kachin State, Myanmar. These included behavior problems, substance use, effects of war, and feeling sad/depressed/hopeless. Descriptions of the problems emphasized the interconnectedness between the problems. For example, substance abuse was linked to behavior problems but also discussed separately, and both were said to result from effects of conflict. Feeling sad/depressed/hopeless was viewed as both a cause and effect of behavior problems and substance use, as well as an effect of conflict. Feelings of being afraid, sad, angry and ashamed were discussed across all problems, and reported as being both the cause and result of inappropriate behaviors such as drugs use. Many of the problems described by respondents appear to be linked with substance abuse, which was reported to be directly related to behavior problems (arguing, lying, stealing**,** bullying and dropping out of school). Substance abuse may also lead to anxiety, insecurity and depression.

Overall, most problems were related to specific events that suggest that the symptoms themselves are responses to unusual situations, such as seeing or experiencing traumatic events from war. However, the problems were also linked to current psychosocial stressors such as poverty, poor nutrition, and stigma and discrimination in the camps. Respondents did report a category of problems described as being effects of war. These were described primarily as a constellation of social and economic problems rather than a list of mental health symptoms, although descriptions of these problems did include post-traumatic stress symptoms. Models describing the impact of mental health in conflict that emphasize the combined impact of trauma exposure and exposure to daily stressors that may be exacerbated by conflict-related conditions have gained increasing support [3]. Additionally, in a review of cultural concepts of distress among conflict-affected groups, Rasmussen and colleagues reported that depression-like syndromes were more commonly reported than PTSD-like syndromes and that there was no clear distinction between descriptions of distress caused by trauma symptoms rather than chronic stressors [22]. Our findings showing little distinction across problem descriptions and an emphasis on causes that include both prior exposure and current psychosocial stressors are consistent with this body of research.

This has implications for intervention design, as it suggests that focusing too narrowly on the impact of trauma exposure alone may fail to adequately address the MHPSS needs of young people affected by conflict. Likewise, the descriptions of problems that highlighted similar symptoms across multiple problem categories suggests that children presenting for treatment may require a blend of intervention strategies focusing on depression, anxiety, problem solving, and trauma recovery. Based on these and similar findings among other trauma-affected populations [16–21], we have focused on adaptation and testing of transdiagnostic treatment approaches that can address not only trauma symptoms but also depression, anxiety, substance use, and problem solving. Use of this approach, CETA, is also supported by a previous study which found an adapted transdiagnostic approach to be effective among adults from Myanmar [14]. CETA was developed to focus on treatment of depression, traumatic stress, and anxiety in low-resource settings by providing engagement, psychoeducation, anxiety management strategies, behavioral activation, cognitive coping, imaginal gradual exposure, as well as components for safety planning (e.g. suicide risk assessment and planning) and substance use reduction [13].

The findings of this have been used to develop and validate a screening instrument and adapt this transdiagnostic intervention for use with this child population. Adaptation of the intervention includes the addition of a parenting component given that data from this study highlight the importance of parents in supporting children. Also, religious leaders, community leaders, and teachers were mentioned as important individuals who currently support children in this context and thus will be informed of the services and asked to encourage youth and caregivers to seek services if they feel they are in need. The current positive coping strategies mentioned by adolescent respondents (e.g. attending church, storytelling, playing music, etc.) will be used to develop the section of the screening instrument that measures daily functioning. In addition, intervention development will also look at how CETA services provided by the local partner organizations might be linked with programs addressing daily stressors identified in the free lists (e.g. early marriage, unwanted pregnancy, school drop out, etc.) given the reciprocal relationship described between these problems and mental health issues.

### Limitations

Data for this study were collected from a convenience sample of adults and adolescents whose views were used to represent common stakeholder perspectives. Children below the age of 10 years were excluded from our study and we instead asked participants to give information on the problems of children as young as 6 years of age. The study is limited to two sites and among beneficiaries from two organizations. However, sites in this study were chosen in partnership with the local organizations and chosen to generally represent the IDP camps, both government and non-government controlled, in Kachin state. Because of this convenience sampling approach, we cannot use the frequency of responses to assume representation of relative prevalence for a given problem within the larger population. In light of this, we instead highlight items reported by more than only one or two people. In fact, in our subsequent experience working with this population, it appears that while the descriptions of the problems described here are accurate, the problems are not highly prevalent.

KI interviews were only conducted in Myitkyina given the security situation in accessing Laiza for this second phase of data collection. However, the FL interviews were conducted in both locations and the situations and language used in both settings are similar. Furthermore, the results from the FL interviews were similar, which suggests that these sites are similar and the KI interview results from Myitkyina likely apply to what would have been found in Laiza. KI interviews were conducted only one time with no follow up interviews; however, the discussions with the data collectors and reviews of the written notes suggest that appropriate probing for responses was done and KI interviewees were unlikely to have given more information in a repeat interview.

## Conclusion

The aim of this study was to gain a better understanding of local perceptions of mental health and psychosocial problems currently impacting Kachin children and adolescents in Myanmar. We also sought to understand how local people perceive the nature of these problems, the effects on individuals and families, as well as coping and support systems for help. Results suggest the common psychosocial problems experienced by displaced young people in Kachin State are a result of both conflict exposure and ongoing daily stressors. Regarding the purpose of this study, findings suggested that our previously developed and tested counseling intervention, the Common Elements Treatment Approach (CETA) [13], would be an appropriate intervention to meet the needs of this population. Problem descriptions have since been used to guide intervention adaptation for children and adolescents in Myitkyina, Myanmar, as well as the development of assessment instruments for use intervention evaluation. Our study also contributes to the limited information available regarding mental health and psychosocial impacts of conflict and displacement on children in Myanmar.

## Abbreviations

AMHR: Applied Mental Health Research group
CETA: Common Elements Treatment Approach
DIME: Design, Implement, Monitor, and Evaluate
FL: Free list
IDP: Internally displaced person
JHU: Johns Hopkins University
KBC: Kachin Baptist Convention
KDG: Kachin Development Group
KI: Key informant
RCT: Randomized controlled trial
